# Recent trends in drugs of abuse metabolism studies for mass spectrometry–based analytical screening procedures

**DOI:** 10.1007/s00216-021-03311-w

**Published:** 2021-04-01

**Authors:** Lea Wagmann, Tanja M. Gampfer, Markus R. Meyer

**Affiliations:** Department of Experimental and Clinical Toxicology, Institute of Experimental and Clinical Pharmacology and Toxicology, Center for Molecular Signaling (PZMS), Saarland University, Kirrberger Straße Building 46, 66421 Homburg, Germany

**Keywords:** New psychoactive substances, Mass spectrometry, Metabolism, Screening, In vitro toxicology, In vivo toxicology

## Abstract

The still increasing number of drugs of abuse, particularly the so-called new psychoactive substances (NPS), poses an analytical challenge for clinical and forensic toxicologists but also for doping control. NPS usually belong to various classes such as synthetic cannabinoids, phenethylamines, opioids, or benzodiazepines. Like other xenobiotics, NPS undergo absorption, distribution, metabolism, and excretion processes after consumption, but only very limited data concerning their toxicokinetics and safety properties is available once they appear on the market. The inclusion of metabolites in mass spectral libraries is often crucial for the detection of NPS especially in urine screening approaches. Authentic human samples may represent the gold standard for identification of metabolites but are often not available and clinical studies cannot be performed due to ethical concerns. However, numerous alternative in vitro and in vivo models are available. This trends article will give an overview on selected models, discuss current studies, and highlight recent developments.

## Introduction

Development and application of analytical screening procedures to identify xenobiotics in human biosamples are amongst the main tasks in clinical and forensic toxicology but also in related fields such as doping control. Methods are often based on mass spectrometry coupled to either gas or liquid chromatography and typical biosamples are blood and urine. To allow reliable identification, analytical data of the screened compounds need to be known. This includes their mass spectra and retention times in a certain setting. To collect this information of parent compounds is often not very challenging as most compounds can be purchased in case of therapeutic drugs or are available from seizures in case of drugs of abuse. Particularly when using urine as matrix for screening, only looking for the parent compound in the sample can lead to false negative results [1]. Many highly lipophilic compounds need to be metabolized before their hydrophilic metabolites can then be renally excreted. In such cases, knowledge about the metabolic fate is essential to screen for the compounds after metabolic transformations in urine [1]. To gain this knowledge, several strategies have been used in the past. Peters and Meyer reviewed in vitro approaches to study the metabolism of new drugs of abuse in 2011 and found that “For identification of a wide variety of phase I and phase II metabolites, intact hepatocytes or liver S9 mix seem most appropriate…” [2]. Particularly over the last decade, further different approaches were investigated to find a suitable model to predict the human metabolism. This was partially accelerated due to the advent of the so-called new psychoactive substances (NPS) over 10 years ago.

Models used for metabolism studies in the field of clinical and forensic toxicology should be easy to handle and cost-effective but should also predict at least the main human urinary excretion products to incorporate them into screening procedures. This trends article aims to summarize the latest development in this field and discuss their pros and cons always with the focus on the requirements of urinary screening procedures. The article will also provide an outlook on upcoming trends.

## Methods

A PubMed-based search for English-written literature using the search term “novel psychoactive substances[Title/Abstract] OR new psychoactive substances[Title/Abstract] AND 2011/01/01[Date - Publication]: 2020/10/29[Date - Publication]” was performed at 29-OCT-2020. In order to identify potential topic related articles and reviews, the results were refined using the additional terms “metabolites”, “metabolism”, “metabolism studies”, or “metabolism models” in all fields. Additionally, scientific literature and reference lists of publications within the scope of the current article were mined to identify relevant, but not PubMed-listed publications.

## Results and discussion

We identified a total of 1387 publications, whereof 215 were review articles. Using the additional term “metabolites” gave 225 results, “metabolism” gave 425 results, “metabolism studies” gave 246 results, and “metabolism models” gave 59 results. Furthermore, four relevant, but not PubMed-listed publications were identified by mining the scientific literature and reference lists of publications within the scope of the current article. As the number of topic related review articles was limited, in particular selected original research articles will be discussed in the following with regard to the scope of the current paper.

## Traditional in vitro and in vivo tools to study the metabolism of NPS

### Human liver microsomes, human liver S9 fraction, recombinant cytochrome P450 enzymes, primary human hepatocytes, and rats

Well-established in vitro systems to study the metabolism of NPS for identification of human biomarkers are human liver microsomes (HLM) and the human liver S9 fraction (HLS9) [2–4]. Such incubations are easily feasible, are cost-effective, and deliver fast results. In comparison to HLM solely containing membrane-bound metabolic enzymes, primarily cytochrome P450 (CYP) and UDP-glucuronosyltransferase (UGT) isozymes, HLS9 further contains soluble enzymes such as *N*-acetyltransferases and sulfotransferases. However, corresponding co-substrates for example of UGT-catalyzed metabolic transformations must be added to the in vitro incubations otherwise potential screening targets are missed. A combination of HLM and human liver cytosol was also tested as an alternative to HLS9 incubations but was not found to provide a benefit [5]. Nevertheless, the authors recommend to use only pooled human liver preparations derived from multiple donors minimizing the influence of interindividual expression differences of metabolic enzymes.

Furthermore, incubations with single recombinant human CYP enzymes are frequently used to identify the isozymes involved in the phase I metabolism of an investigated compound [6, 7]. Such information can be useful for predicting drug-drug or drug-food interactions as well as the influence of polymorphisms leading to interindividual activity differences of a metabolic enzyme.

In 2011, Peters and Meyer found that primary human hepatocytes (PHH) were only scarcely used to investigate the metabolism of new drugs of abuse [2], but in the meantime further studies were published [3, 8]. Although PHH are regarded as an excellent alternative for metabolism studies of NPS compared to human biosamples, high purchase costs and unstable CYP enzyme expression are regarded as main limitations [3].

Concerning in vivo approaches, rats were the most commonly used mammalian model to identify putative NPS biomarkers. Usually, their urine was collected over a defined time period allowing the identification of early- and late-phase excretion products. Rats are affordable and simple in handling but keeping of animals causes high costs and an ethical approval for animal experiments is required. Furthermore, species differences in expression of metabolic enzymes and thus in the metabolic pattern of the investigated drug of abuse should be considered [3].

In the following sections, trends for studying the metabolism of NPS with focus on developing mass spectrometry–based screening procedures observed over the last decade will be discussed. First, in vitro approaches will be described and the conditions of selected studies are summarized in Table 1. Then, in vivo tools other than humans are going to be presented (summary in Table 2). Individual pros and cons are going to be discussed (overview in Table 3) and finally the metabolites detected in the model systems will be compared to human data if available.

**Table 1:** Summary of selected studies using in vitro models identified as trends for studying the metabolism of NPS along with several parameters such as investigated NPS, experimental setup, time requirement, and financial costs

In vitro model	Literature	Investigated NPS	Experimental setup	Time requirement	Financial costs
Recombinant phase II isozymes					
UDP-glucuronosyltransferases	Pettersson Bergstrand et al., 2019 [9]	9 designer benzodiazepines	Incubation with 13 different isozymes (60 min, 37 °C)	<1 day	+
	Wagmann et al., 2020 [10]	Bromazolam, clobromazolam	Preincubation with CYP3A4 (60 min, 37 °C) and incubation with 13 different isozymes (120 min, 37 °C)		
Subcellular fractions					
Pig liver microsomes	Nordmeier et al., 2020 [11]	U-47700	Incubation for 30 min (37 °C)	<1 day (+ previous isolation from pig liver and determination of protein amount)	+
Cellular systems					
HepG2 and HepaRG	Richter et al., 2017 [5]	6 methylenedioxy derivates and 2 bioisosteric analogs	Incubation for 24 h (37 °C, 95% air humidity, 5% CO_2_), analysis of supernatant	2 days (+ previous cultivation, HepG2: 3 days, HepaRG: 6 days)	++
HepaRG	Richeval et al., 2017 [12]	Furanylfentanyl	Incubation for 6, 24, or 48 h (37 °C with 5% humidified CO_2_), analysis of supernatant	3 days (+ 4 weeks for cultivation)	++
	Wagmann et al., 2020 [10]	3,4-DMA-NBOMe, ephylone, 4F-PHP,1-propionyl-LSD, 4F-MDMB-BINACA	Incubation for 24 h (37 °C, 95% air humidity, 5% CO_2_), analysis of supernatant	2 days (no previous cultivation, only 4 h for cell adhesion)	
Hepatocytes of mice with a humanized liver	Kanamori et al., 2018 [13]	Acetylfentanyl	Incubation for 24 or 48 h (37 °C, 5% CO_2_), analysis of medium	3 days (+ 4 or 11 days for cultivation)	+++
Zygomycetes					
*Cunninghamella elegans*	Watanabe et al., 2017 [14]	5F-PB-22, PB-22, XLR-11, UR-144	Analysis of filtrate after incubation (72 h, 26 °C)	4 days (+ 7 days for cultivation)	++
	Grafinger et al., 2019 [15]	DMT, 4-HO-MET, 5-MeO-DALT, 5-MeO-MiPT	Analysis of growth medium and fungi biomass after incubation (72 h, 30 °C)

**Table 2:** Summary of selected studies using in vivo models identified as trends for studying the metabolism of NPS along with several parameters such as investigated NPS, experimental setup, time requirement, and financial costs

In vivo model	Literature	Investigated NPS	Experimental setup	Time requirement	Financial costs
Mice with a humanized liver	De Brabanter et al., 2013 [16]	JWH-200	Oral administration, urine collection after 24 h and 48 h	3 days	+++
Pig	Walle et al., 2021 [17]	Cumyl-5F-P7AICA	Pulmonary administration, urine collection in 1 h intervals for 8 h	1 day	+++
	Nordmeier et al., 2020 [11]	U-47700	Intravenous administration, urine collection in 1 h intervals for 8 h		
Zebrafish	Sardela et al., 2018 [18]	JWH-073	Adult zebrafish (3–5 months) exposure via tank water (28 °C), tank water collection after 0, 3, 6, 24, 48, 72, 96, 120, 144, and 168 h	> 1 week	++
	Wagmann et al., 2020 [10]	3,4-DMA-NBOMe, ephylone, 4F-PHP,1-propionyl-LSD, 4F-MDMB-BINACA	Exposure to larvae (4 days post-fertilization) via medium (24 h, 28 °C), analysis of larvae after extraction	2 days

**Table 3:** Summary of advantages and disadvantages of in vitro and in vivo metabolism models identified as trends for identification of NPS screening targets: CYP, cytochrome P450

Model	Advantages	Disadvantages
In vitro		
UDP-glucuronosyltransferases	Fast, simple	Additional preincubation step required if substrate is a phase I metabolite
HepG2	Cost-effective High proliferation rate	Low gene expression of several CYP enzymes ➔ Poor overlap with human data Special equipment and trained staff for cell culture required
HepaRG	Cost-effective Generate many metabolites	Low gene expression of CYP2D6 Special equipment and trained staff for cell culture required
Hepatocytes of mice with a humanized liver	Cell culture over several weeks feasible	Expensive Contaminated with murine hepatocytes Special equipment and trained staff for cell culture required
Fungus *Cunninghamella elegans*	Simple	Potential species differences Special equipment and trained staff for fungal culture required
In vivo		
Mice with a humanized liver	Offer a humanized liver	High costs Remaining murine hepatocytes and potential species differences Ethical approval for animal experiment required
Pig	Produce many metabolites	Special equipment and trained staff for surgical procedure required Potential species differences Ethical approval for animal experiment required
Zebrafish larvae	No animal experiment until 5 days post-fertilization in EU Produce many metabolites	Experience in zebrafish breeding and special equipment required Potential species differences
Zebrafish	Holds several direct human CYP orthologs Produce many metabolites	Ethical approval for animal experiment required Experience and special equipment required Potential species differences

## In vitro trends for studying the metabolism of NPS

### Recombinant isozymes for studying phase II metabolism: UGT

As liquid chromatography–based screening procedures became more and more popular in recent years, the importance of phase II metabolites, particularly glucuronides and sulfates, as additional bioanalytical targets for drugs (of abuse) also increased. However, their use, particularly as urine screening targets, strongly depends on the sample preparation, which must contain neither mineral (i.e., acidic and alkaline) nor enzymatic conjugate cleavage. Several studies used single enzyme incubations with recombinant human UGT isoforms for identification of screening targets and involved isozymes [6, 9, 10, 19]. Some designer benzodiazepines were found to undergo direct glucuronidation of the parent compound as well as glucuronidation of suitable phase I metabolites. The latter was also true for *N*-methoxybenzyl (NBOMe)-containing NPS. The phase I metabolites suitable for conjugation were produced by preincubation of the NPS with a single CYP isozyme previously found to catalyze the phase I metabolic reaction of interest [6, 10, 19]. After preincubation with CYP3A4, Wagmann et al. detected two out of three flualprazolam glucuronides in UGT incubations compared to pooled HLS9 incubations [6]. This in vitro approach was further improved using a longer CYP3A4 preincubation and higher enzyme concentrations allowing the detection of all bromazolam and clobromazolam glucuronides simultaneously detected in positive control incubations with pooled HLM [10]. Pettersson Bergstrand et al. detected parent glucuronides of seven out of nine designer benzodiazepines in in vitro incubations and identified UGT1A4 as the main catalyzing isozyme [9].

### Cellular systems: HepG2, HepaRG, hepatocytes of mice with a humanized liver

The human hepatic cell lines HepG2 and HepaRG, both derived from patients suffering from liver carcinoma, were recently tested as alternatives to PHH with the aim to overcome their disadvantages such as high purchase costs and unstable CYP enzyme expression. However, it should be considered that all cell culture experiments require special laboratory equipment as well as trained personnel. Unfortunately, HepG2 cells are known for their poor gene expression of several CYP enzymes, which might explain the low number of publications in the context of NPS metabolism studies [5, 20, 21]. HepaRG cells are more frequently used to identify metabolites of NPS, probably due to the fact that HepaRG cells show gene expression levels for metabolizing enzymes comparable to PHH. The only exception is CYP2D6 as the donor of the HepaRG cell line was found to have a low CYP2D6 gene expression.

HepG2 cells were described to be able to form the most abundant metabolites of the opiate desomorphine in comparison to rat urine [20]. However, HepG2 cells produced far less metabolites of eight NPS (six methylenedioxy derivates and two bioisosteric analogs) than pooled human liver preparations and HepaRG cells in a comparative work performed by Richter et al. later on [5]. Richeval et al. determined all furanylfentanyl metabolites in HepaRG cell incubations previously identified in HLM incubations plus nine additional metabolites [12]. HepaRG cells were also used by Wagmann et al. evaluating and comparing the metabolic fate of five NPS from different classes in two different in vitro and one in vivo model. The study included the amphetamine-based compound 3,4-DMA-NBOMe, the synthetic cathinone ephylone, the pyrrolidinophenone analog 4F-PHP, the lysergamide 1-propionyl-LSD, as well as the synthetic cannabinoid 4F-MDMB-BINACA. Overall, HepaRG cells were found to produce more phase I and II metabolites than pooled HLS9 [7].

Furthermore, hepatocytes freshly isolated from liver-humanized mice can be used for in vitro studies. In such chimeric mice, a high amount of the murine hepatocytes was replaced with human hepatocytes. Stable activity of drug-metabolizing enzymes allows to cultivate and store these cells over several weeks. However, such hepatocytes are more expensive than cell lines and are contaminated with up to 10% of murine hepatocytes [22]. Two fentanyl derivates were investigated in such an in vitro setting by Kanamori et al. with focus on identification of phase I metabolites after conjugate cleavage [13, 23]. A comparison of acetylfentanyl metabolites with those formed in PHH revealed good agreement [13].

### Zygomycetes: *Cunninghamella elegans*

The fungus *C. elegans* represents a microorganism-based model for studying the metabolism of NPS. It is less expensive and easier to handle than the aforementioned cell lines, but drug exposure takes several days and special equipment and personnel experienced in microbial culture are required. *C. elegans* is known to have enzymatic activity for both phase I and II reactions with some similarities in CYP enzyme expression but equal differences especially in phase II enzymes compared to humans. Watanabe et al. and Leong et al. described that *C. elegans* was able to generate both phase I and II metabolites in case of four out of five synthetic cannabinoids [14, 21]. By contrast, Grafinger et al. detected only phase I metabolites of four investigated synthetic tryptamines in fungi biomass and growth medium used [15]. However, it should be considered that different fungus strains were used. The review by Diao and Huestis summarized the pros and cons of fungus *C. elegans* but also further model systems for identification of synthetic cannabinoid biomarkers and is recommended to interested readers [3].

## In vivo trends for studying the metabolism of NPS

### Chimera: mice with a humanized liver

As controlled trials with humans involving large patient populations to investigate the metabolic fate of NPS are hardly feasible due to potential toxicity and ethical concern, alternative in vivo metabolism models may be used. Unfortunately, species differences influencing metabolic transformations are regularly observed. By replacing a high number of murine hepatocytes with human hepatocytes, chimeric mice with a humanized liver are created. These animals are intended to overcome species differences as chimeric mice offer a highly humanized liver with stable human hepatocyte engraftment expressing human drug-metabolizing enzymes [22]. The procedure of humanization is laborious, humanized mice are expensive, and an ethical approval is required. Furthermore, up to 30% murine hepatocytes remain present and metabolic transformations also occur in other (murine) organs meaning that species differences are still possible. De Brabanter et al. published two studies investigating the metabolism of the two synthetic cannabinoids JWH-200 and JWH-122 [16, 24]. Urine of chimeric mice after oral administration was used as in vivo confirmation of the metabolites identified in vitro using HLM incubations. The authors were able to confirm most of the metabolites identified in vitro and stated that all metabolites formed in vivo were excreted as glucuronide or sulfate, partly with conjugation rates above 50%. Furthermore, non-chimeric mice were used as control group to identify metabolites most likely produced by the human hepatocytes [16, 24].

### Pig

Metabolism studies with NPS were performed in pigs as they were previously shown to be suitable for modeling the toxicokinetics of THC. Pigs were described to be closely related to humans in terms of metabolism including CYP enzyme pattern, anatomical structures, and physiological properties [25]. Domestic pigs (Swabian Hall strain) were used to elucidate the metabolic fate of selected synthetic cannabinoids and opioids usually using urine as sample matrix [11, 17, 26]. Some studies were performed using intravenous administration, but as synthetic cannabinoids are usually smoked by human consumers, a method for pulmonary administration was applied using e.g. cumyl-5F-P7AICA and 5F-MDMB-P7AICA (also known as 7′N-5F-ADB) [17, 26]. Furthermore, pig liver microsomes were produced by the authors and used as additional in vitro model for comparison with in vivo data [11, 17, 26]. Pigs were found to produce multiple metabolites and the authors therefore assessed pigs as suitable in vivo metabolism model for NPS. Nevertheless, keeping of animals is expensive, ethical approval and personnel experienced in surgical procedures are mandatory, and species differences cannot be excluded.

### Zebrafish and zebrafish larvae

Zebrafish (*Danio rerio*) has become a popular metabolism model organism with a drug metabolism expected to be similar to that of mammals as several metabolic enzymes in zebrafish have direct orthologs in humans. The zebrafish is a small teleost typically from sweet waters that can be easily maintained with a high reproductive rate [3]. However, establishing and maintaining a zebrafish culture platform is expensive and experienced personnel are required. Not only the adult zebrafish but also zebrafish larvae were used to generate NPS metabolites. The larvae provide all benefits of intact organisms such as (re)absorption, distribution, and excretion processes but are not considered as animals until 5 days post-fertilization according to the European Directive 2010/63/EU. Consequently, zebrafish larvae experiments do not require an ethical approval in contrast to zebrafish experiments in the EU if they are completed within 5 days after fertilization.

Richter et al. used the synthetic cannabinoid 5F-MDMB-P7AICA as a model compound to investigate the potential of the zebrafish larvae model as a preclinical surrogate for the human hepatic metabolism of NPS to develop toxicological screening procedures [27]. The authors compared different experimental protocols for zebrafish larvae exposure such as addition of 5F-MDMB-P7AICA to the culture medium or application by microinjection into the yolk sac and analysis of the larvae or the surrounding culture medium. As almost no metabolites could be detected after microinjection into the yolk sac, they assessed the incubation via medium and the analysis of the extracted zebrafish larvae as the most suitable experimental protocol. Furthermore, the metabolites detected in the zebrafish larvae were compared to those detected in in vitro incubations using pooled HLS9 but also HepaRG cells. Zebrafish larvae and HepaRG cell incubations were found to provide the highest number of metabolites and the authors concluded that zebrafish larvae may be a promising model for studying the toxicokinetics of NPS, but further studies comparing different NPS classes were needed [27]. In a follow-up study, Wagmann et al. [7] compared the metabolic fate of five NPS from different classes (3,4-DMA-NBOMe, ephylone, 4F-PHP, 1-propionyl-LSD, 4F-MDMB-BINACA) in a similar setting. Zebrafish larvae were found to produce the highest number of phase I but also phase II metabolites (79 metabolites in total), followed by HepaRG cells (66 metabolites), and pooled HLS9 (57 metabolites). However, the findings by Richter et al. [27] indicated that the route of application influences the experimental outcome and Park et al. recently confirmed this assumption and detected a high number of 5F-MDMB-P7AICA metabolites in zebrafish larvae after microinjection into the caudal vein, heart ventricle, or hindbrain [28].

Adult zebrafish (3–5 months) were used by Sardela et al. to investigate the metabolic fate of JWH-073 [18]. Application of the synthetic cannabinoid was performed via the tank water and 5-mL aliquots were collected for analysis at a total of 10 different time points over 7 days. The samples were treated with β-glucuronidase before solid-phase extraction. Two monohydroxylated metabolites were detected with the hydroxy group located at the butyl chain in both cases. In the last days of the experiments, a follow-up metabolite formed by oxidation to the carboxylic acid was described to be the main metabolite present in the tank water.

## Comparison with human data

If clinical or forensic toxicologists intend to detect the intake of NPS by analyzing human biosamples, chromatographic and mass spectral information of metabolites collected during metabolism studies must be implemented into screening procedures. This is particularly important in case of urine screening [1]. The implementation should not be motivated by the abundance of the metabolite as a minor metabolite in a non-human system may be a major metabolite in humans [7]. Vice versa, a high number of metabolites identified using a metabolism model system alone does not necessarily mean that this model system is the most suitable one for developing analytical procedures for human biosamples as the detection of NPS can only be successful if the implemented metabolites will also be present in human biosamples [7]. To assess the suitability of novel in vitro and in vivo models for the prediction of NPS screening targets in human biosamples, the metabolites detected in the model systems should always be compared to recommended screening targets in human biosamples if available. However, it should be kept in mind that the investigation of single NPS or NPS classes has only limited conclusiveness.

Concerning the cellular systems in general, the metabolites generated by HepG2 cells provided the smallest overlap with human data [5, 21]. Metabolites formed by the fungus *C. elegans* showed a moderate or good agreement with confirmed human biomarkers [14, 21]. The metabolic fate of the synthetic cannabinoid 5F-MDMB-P7AICA was extensively investigated in different in vitro and in vivo model systems as well as in human biosamples [26, 27, 29]. Six 5F-MDMB-P7AICA metabolites were identified as most abundant signals in a total of six human urine samples [29]. Three of them were detected in pooled HLS9 incubations, four in zebrafish larvae, and five in HepaRG cell incubations. Richter et al. concluded that zebrafish larvae and HepaRG cell incubations provided the most comprehensive spectrum of human urinary metabolites, but a successful detection should also be possible based on HLS9 incubations [27]. Doerr et al. also compared their pig model–based findings with the human metabolites and described a similar metabolite pattern in pig urine [26, 29]. Nordmeier et al. also concluded that the metabolic pattern of the synthetic opioid U-47700 elucidated in the pig model was comparable to human in vivo data [11]. Wagmann et al. compared their findings with the most abundant signals detected in human plasma and urine after consumption of ephylone, 4F-PHP, or 4F-MDMB-BINACA, as no human data after intake of 3,4-DMA-NBOMe and 1P-LSD was available. Zebrafish larvae experiments (92%) agreed best with human data, followed by pooled HLS9 incubations (88%) and HepaRG cell experiments (79%) [7].

## Summary

During the last decade, several trends in drugs of abuse metabolism studies for mass spectrometry–based analytical screening procedures could be identified covering both in vitro and in vivo model systems. As expected, each model provides individual strengths but also weaknesses, which must be considered beforehand. Nevertheless, the most convenient tools used in clinical and forensic toxicology are still incubations with human liver preparations such as HLM and HLS9 as they are easy to apply, cheap, and deliver fast results. In most cases, such incubations are expected to be an appropriate tool for the identification of metabolites of NPS in order to develop mass spectrometry–based bioanalytical screening procedures. However, alternative model systems may provide a higher number of metabolites allowing to assess the metabolic fate of a drug of abuse more comprehensively. In vivo tools may also allow to investigate further aspects of their toxicokinetics such as absorption, distribution, or excretion processes.

## Outlook

Reduction and replacement of animal experiments are recommended throughout the scientific disciplines for ethical reasons. This motivation leads to the continuous advancement of in vitro models, which are expected to represent the future for metabolism studies. The main aim is to overcome common disadvantages of in vitro model systems used to investigate the metabolism of exogenous compounds such as the limited expression of enzymes, the lack of drug disposition, and the limited viability. As the liver is the main organ for drug metabolism in humans, incubations with liver cell preparations are widely used, but not the full spectra of enzyme-catalyzed reactions occurring in the human liver are observable. The microsomal fraction only contains membrane-bound enzymes, while the cytosol only contains soluble enzymes. The HLS9 combines both enzyme groups, but only intact cells additionally express transport proteins. Nevertheless, as experiments are usually performed in an enclosed experimental environment, influence of absorption, distribution, and excretion processes cannot be considered. Therefore, microfluidic cell culture platforms, for example, the so-called organs-on-a-chip, may be described as innovations in the field [30]. Organs-on-a-chip are a type of artificial organs formed by a multi-channel 3D microfluidic cell culture chip that simulates the activities, mechanics, and physiological response of entire organs and organ systems. Having said that, high costs and prerequisites such as laboratory equipment and skilled personnel currently limit the application of these innovative techniques in the field of clinical and forensic toxicology. Furthermore, the validation of novel in vitro metabolism models takes time as comprehensive testing is required before routine or even high-throughput application.
